# Equalizing the response to AIDS and other pandemics

**DOI:** 10.1371/journal.pgph.0001370

**Published:** 2022-12-01

**Authors:** Winnie Byanyima, Matthew M. Kavanagh

**Affiliations:** 1 United Nations Joint Programme on HIV/AIDS (UNAIDS), Geneva, Switzerland; 2 Georgetown University, Washington, D.C., United States of America; PLOS: Public Library of Science, UNITED STATES

The Global AIDS response is in danger. Colliding HIV and COVID-19 pandemics, war, and economic and humanitarian crises have knocked the AIDS response off course and deepened some of the key inequalities driving the pandemic. New HIV infections are rising in Latin America, Eastern Europe and Central Asia, the Middle East and North Africa. New infections are now increasing in Asia, where they had been falling, and while still declining in Eastern and Southern Africa, progress is slowing [1]. These colliding crises also threaten the world’s capability to respond effectively to other current pandemics, and to prepare for the pandemics to come.

Moments of crisis can create political opportunities for change [2, 3]. In this case, a shift toward tackling AIDS by addressing inequalities head-on is the only way out of danger. Doing so could enable world leaders to end AIDS as a global public health threat by 2030 as promised. This equalizing approach will also provide a path to effectively tackle other pandemics.

Tackling inequalities requires more than recognizing disparities and those “left behind.” It necessitates identifying and addressing power [4]. The bigger the gap in power between those whose needs are met and those whose needs are not, the more difficult it is to end a pandemic. For millions, the AIDS response has been highly effective—AIDS deaths have fallen by 52% since 2010 [5] and many people today face greatly reduced risk of acquiring HIV and of dying if they do. But despite remarkable biomedical innovations, approximately 1.5 million new HIV infections occurred last year—more than 1 million more than the global targets [1]. 7 million people are likely to die in the next ten years on our current trajectory [6]. Success for some—particularly those who are most visible and politically powerful—has made it easier to ignore those for whom AIDS remains a crisis. In a pandemic, invisibility provides a foothold for viruses to spread.

There is ample opportunity to take action to shrink inequalities at the local, national, and international level. Here we give four examples.

We start by addressing the inequalities driving disproportionate HIV infection and death among key populations. Gay men and other men who have sex with men have 28 times greater risk of acquiring HIV than adult men (15–49) in the general population [1]. People who inject drugs have 35 times greater risk of acquiring HIV than adults who do not. Female sex workers a 30 times greater risk than adult women in the general population. Transgender women have 14 times the risk of other adult women. Expanding services for these populations is incredibly important, but insufficient. Each of these unequal outcomes is driven by political decisions—in law, policy, and health programming. Punitive and discriminatory laws and policies criminalizing these populations undermine the AIDS response by pushing people away from services and undermining public health efforts to reach those most at risk. Indeed, countries that criminalize key populations have made less progress on ensuring that people living with HIV know their status and are on effective antiretroviral therapy than those that avoid criminalizing approaches [7]. In June 2021, UN member states committed at the High Level Meeting on HIV/AIDS to removing these laws and creating legal environments that better enable pandemic action—setting a goal that by 2025 less than 10% of countries would have restrictive law and policy frameworks that push people away from services [8]. And there is progress. Recently, countries from Angola to Saint Kitts & Nevis to Singapore have announced the removal of laws that criminalize gay relationships, for example.

We can also address gender-based HIV inequalities. Adolescent girls and young women (aged 15 to 24 years) are three times more likely to acquire HIV than adolescent boys and young men of the same age group in sub-Saharan Africa [1]. While important progress has been made, these inequalities for girls have not been closed by the many good prevention programs. We must ask, then, how to interrupt the power dynamics that make girls more vulnerable. Secondary education may provide a critical tool. Evidence shows that, for girls, further education increases their earnings, social inclusion, and decision-making power [9]. It also increases their knowledge of HIV and, as several studies from African countries suggest, reduces their risk of acquiring HIV [10]. When Botswana extended mandatory secondary education, for instance, it found that each additional year of schooling after Year 9 was associated with a 12% reduction in girls’ risks of acquiring HIV [11].

In addition to actions that can close inequalities between populations in a local context, actions are also needed to tackle the international inequalities which drive the AIDS pandemic.

Equitable access to innovative medical technologies, for example, can help stop pandemics. While unequal COVID-19 vaccine access made headlines, another case in point is long-acting antiretrovirals for HIV. New long-acting cabotegravir (CAB-LA) for pre-exposure prophylaxis (PrEP) provides eight weeks of continuous protection against HIV infection through a single intramuscular injection. The World Health Organization has recommend CAB-LA for use worldwide after randomized controlled trials comparing long-acting injectable PrEP to oral PrEP found higher adherence and a remarkable 79% reduction in the risk of HIV acquisition among those using the injectable compared to taking a pill every day [12, 13]. For some, this could be a game-changer—enabling them to avoid challenges of taking a pill every day or the stigma of being seen doing so. Young women living with their parents, gay people or sex workers worried about criminalization, those with insecure housing—particularly people in the global South could benefit. It could be a tool to reduce inequality.

**Fig 1:**
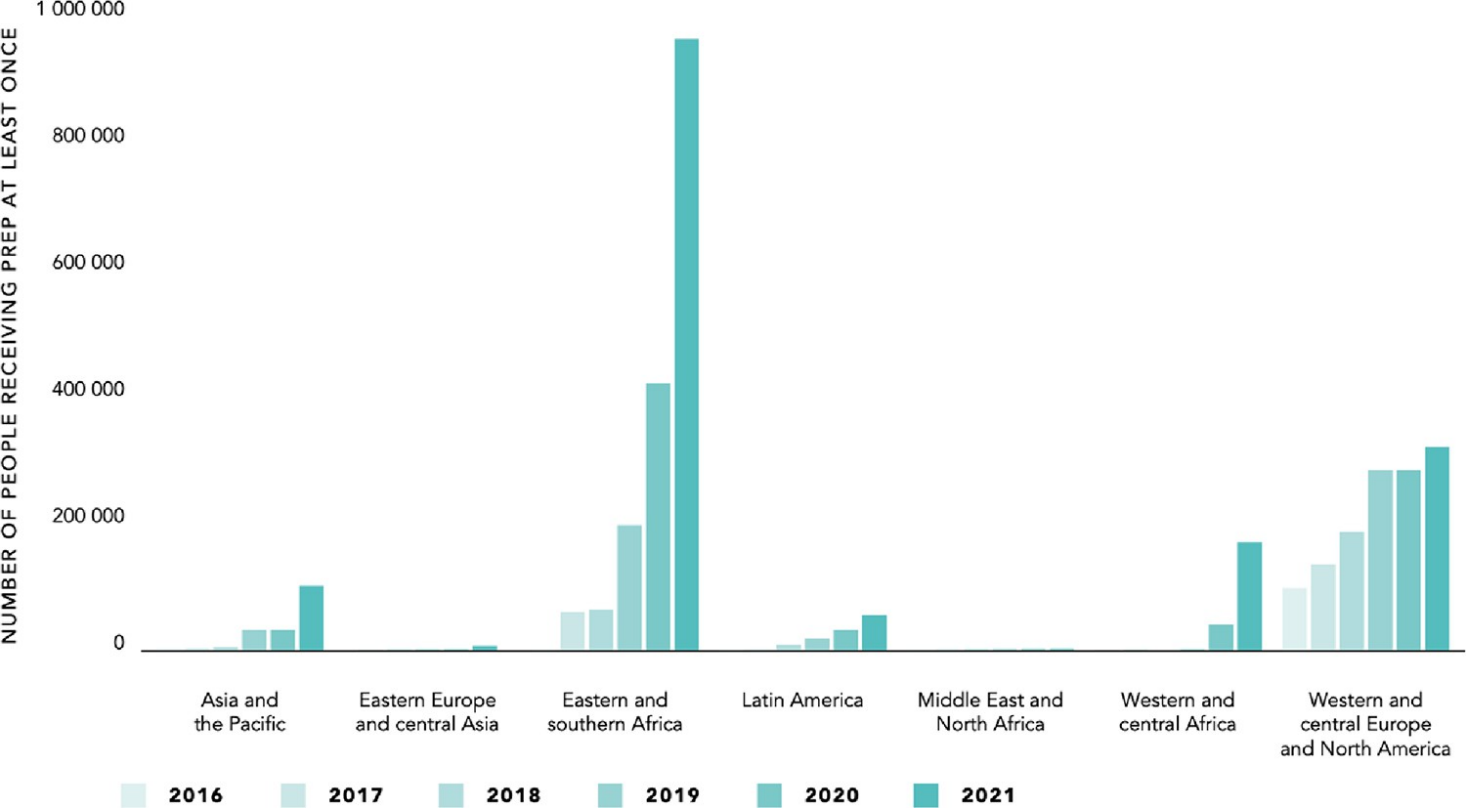
Number of people who received pre-exposure prophylaxis (PrEP) at least once during the reporting period, by region, 2017–2021 (Source: UNAIDS 2022).

Equal access is especially important since oral PrEP has, to date, been a driver of inequality. From its first approval in 2012 until 2020 there were more people accessing PrEP in Europe and North America than in all of Africa (Fig 1). That nearly decade-long unequal head start led to remarkable declines in HIV rates, especially in some key high-income cities [14], but provided little benefit to the rest of the world. So far, long-acting PrEP is rolling out in high income cities like New York and London, but is nearly unavailable in Africa, Asia, and Latin America due to a price that is unaffordable for low- and middle-income countries. This price barrier will further exacerbate global inequalities. Governments and international institutions have a strong role to play here—pushing to ensure that the maker of Cabotegravir reduces the price further and that technology is quickly transferred so lower-cost generic versions produced in low- and middle-income come to market rapidly. Better yet would be taking lessons on what has worked and not for efforts like the medicines patent pool and the new COIVD-19 technology transfer hub in South Africa. Crafting a global agreement to share pandemic-fighting technologies and support rapid production in hubs around the world, would be better than addressing this drug by drug, disease by disease.

International financing, too, can drive or reduce inequalities. During COVID-19 high-income countries invested billions in their economies and health systems—avoiding the worst of a possible fiscal crisis. Many low- and middle-income countries have had limited capacity to do the same. Those who did invest now face a growing debt crisis that is squeezing out spending to fight AIDS, pandemics, and to ensure universal health. UNAIDS’ estimates that in countries with high rates of HIV and high debt burden, for every 10 dollars of government revenue, four were allocated to debt service and only one to health in 2020 [15]. 37 countries with the highest levels of debt spent more resources on debt service than health in the middle of the COVID-19 pandemic in 2020. This should tell us that the international financial institutions need to take an urgent look at priorities to support wellbeing in this time of crisis. Rapid debt relief and additional aid are both needed to equalize fiscal space to end AIDS.

Viruses thrive on inequalities. Until we tackle the inequalities driving AIDS, we cannot end it, and we risk resurgence.

Tackling these and other critical areas of inequality matter for ending the AIDS pandemic as well as for other pandemics. Monkey Pox remains a declared Public Health Emergency of International Concern for which the effective vaccines are unavailable in many low- and middle-income countries; Criminalization and exclusion continues to make some communities more vulnerable to the continuing COVID-19 pandemic. And inequalities in fiscal space are undermining capacity for many countries to prepare for the pandemics to come.

One hopeful lesson from history is that it has often been in times of crisis that leaders have found the opportunity to make long-overdue transformative change. Solutions are not mysteries. We know them. The challenge is political. With growing attention to the ways inequalities drive pandemics, we have a window of opportunity in which to build rights-based, human-focused responses to save millions of lives, beating the pandemics raging today and building a pandemic-resilient for the future. But this is possible only if leaders *seize* that opportunity, by working courageously and together to tackle the inequalities endangering us all.
